# Effect of Multiple Parasitic Infections on the Tolerance to Pollutant Contamination

**DOI:** 10.1371/journal.pone.0041950

**Published:** 2012-07-26

**Authors:** Eric Gismondi, Thierry Rigaud, Jean-Nicolas Beisel, Carole Cossu-Leguille

**Affiliations:** 1 Laboratoire des Interactions Ecotoxicologie Biodiversité Ecosystèmes (LIEBE), Université de Lorraine (UdL), Campus Bridoux, Bât. IBiSE, Metz, France; 2 Equipe Ecologie Evolutive, Biogeosciences, Université de Bourgogne, Dijon, France; Universite de la Mediterranee, France

## Abstract

The horizontally-transmitted acanthocephalan parasite *Polymorphus minutus* and the vertically-transmitted microsporidian parasite *Dictyocoela roeselum* have both been shown to influence on the antitoxic responses of mono-infected *Gammarus roeseli* exposed to cadmium. The present study investigates the effect of this co-infection on the antitoxic defence responses of naturally infected females exposed to cadmium stress. Our results revealed that, depending on the cadmium dose, bi-infection induced only slight, significant increased cell damage in *G. roeseli* as compared to non-infection. In addition, the antitoxic defence pattern of cadmium-exposed bi-infected hosts was similar to the pattern of cadmium-exposed *D. roeselum*-infected hosts. Reduced glutathione concentrations, carotenoid levels and γ-glutamylcystein ligase activity decreased, while metallothionein concentrations increased. This similar pattern indicates that host physiology can be controlled to some extent by microsporidia under stress conditions. It supports the hypothesis of a disruption of acanthocephalan effects in the presence of microsporidia. However, the global negative effects of bi-infection on host condition should be tested on more biological models, since competition between parasites depends on life history trade-off.

## Introduction

Aquatic environments are more and more contaminated by human activities, and these disturbances are now well known to cause dysfunctions in organisms. To cope with pollutant-induced stress, organisms have developed antitoxic defence capacities, which are essential for keeping them alive in contaminated environments and thereby, for maintaining their fitness [1], [2].

In the last decade, the influence of parasites on host physiology has increasingly been studied in the context of contaminated environments. Previous studies have highlighted a disruption of behavioural and biological host responses due to the presence of parasites [3], [4]. Indeed, in a contamination context, host antitoxic responses can increase [5] or decrease [6] as a result of parasitic infection, and therefore lead to differential host sensitivity [7]. However, these observations were often obtained with infections by single parasite, but field conditions are quite different. Indeed, most living species are infected by parasite assemblages, and it is quite common for individual hosts to harbour multiple parasite species (e.g. [8], [9]). The presence of two parasitic species co-infecting the same host could lead to competition or conflicting situations that could influence parasite virulence [10] or host resistance [11]. To our knowledge, no study has yet been devoted to investigating the effect of a bi-parasitic infection on host antitoxic defence capacities.

Gammaridae (Crustacea, Amphipoda) are increasingly used as a biological model for assessing contamination in freshwater ecosystems, mainly because of their key role and widespread distribution [12]. They are nevertheless also well known for getting infected by numerous parasites, and multiple infections have been reported repeatedly [13], [14]. *Gammarus roeseli* is the intermediate host of various acanthocephalan parasites, whose final hosts are fish or water birds [15], [16]. Acanthocephalans are known to alter the behaviour of their intermediate host in a way that makes it more prone to predation and thus favours its transmission to the final vertebrate host [17], [18]. Behavioural changes induced by acanthocephalans are varied and include reaction to light [13], [19], vertical distribution [20], drift behaviour [21], [22], activity levels [23], or refuge use and escape performance [24]–[26]. Acanthocephalan parasites can also influence on their host's antitoxic defences [27]. *Polymorphus minutus*, an acanthocephalan bird parasite, is also known to castrate its intermediate amphipod host [28]. This trait, added to parasite-induced increased predation by water birds, clearly depicts *P. minutus* as harmful to its host. *G. roeseli* is also the host of various vertically-transmitted microsporidia parasites that are egg-transmitted from females to their offspring [7], [29]. A conflicting situation is predicted when vertically-transmitted parasites and obligate horizontally-transmitted parasites co-occur in the same individual host [30]: acanthocephalans use intermediate hosts for their transmission to the final host via the trophic chain, which results in host death, while microsporidia totally rely on their gammarid host's survival and reproduction for their transmission. In nature, microsporidian infection always precedes acanthocephalan infection, due to their transmission way. Super-infection by *P. minutus* is therefore clearly unfavourable, and leads to a potential conflict between the two parasites.

However, microsporidia parasites do not prevent co-infection by *P. minutus*. Rather, they induce a decrease of the behavioural manipulation exerted by the acanthocephalan [14]. A theoretical study showed that a virulent vertically-transmitted parasite could persist by protecting the host from horizontally-transmitted parasite virulence, especially if the latter was a castrating parasite [31]. However, no study has yet been devoted to investigating the consequences of the presence of vertically- and horizontally-transmitted parasites sharing the same host and exposed to a chemical stress. *P. minutus* and microsporidia are both known to disrupt *G. roeseli* antitoxic defences in the case of mono-infections. Microsporidia have no major impact on their host in unstressed conditions, but their presence increases host cell damage after moderate cadmium exposure and induces a slight negative impact on antitoxic defences [7]. Similarly, we showed that *P. minutus* increased cell damage in *G. roeseli* in cadmium exposure, although the antitoxic defence capacities were increased in infected individuals (unpublished data).

From these works, and from the former studies on multiple infections described above, predictions about the outcome of co-infection in cadmium-exposed *G. roeseli* could be two-fold: either co-infections strongly weakens hosts and thus increases their sensitivity to the pollutant because co-infection increases the virulence or, conversely, co-infection causes lower disturbances as compared to infection by *P. minutus* alone, thanks to the protection that microsporidia confer to their hosts.

In this work, we tested those two hypotheses by studying the influence of the co-occurrence of *P. minutus* and of the microsporidia *Dictyocoela roeselum*, on the energetic reserves and antitoxic defences of naturally infected *G. roeseli* females, under a cadmium stress (see Materials and Methods section). Antitoxic defence capacities were studied by assaying several markers: concentrations of reduced glutathione (GSH), a tripeptide that plays an essential role in the detoxification system by scavenging organic or metallic xenobiotics thanks to its thiol group [1] and being substrate of several antitoxic enzyme glutathione-dependent (i.e. glutathione-S-transferase, glutathione peroxidase); the activity of γ-glutamylcysteine ligase (GCL, EC 6.3.2.2), the limiting enzyme of *de novo* GSH synthesis; concentrations of metallothioneins (MT) which are involved in binding metallic compounds and contribute to protecting tissues against oxidative damage [32], [33]; and carotenoid levels, which are involved in reproduction [34] and in antioxidant defences [35]. In parallel, levels of malondialdehyde (MDA), a product of the lipoperoxidation considered as a toxic effect biomarker, were also measured. Moreover, energy reserves were estimated by measuring total lipid and glycogen contents. Glycogen levels are representative of the energy available for current activities [36] whereas lipids are stored in fat bodies and are used during starvation or reproduction periods [37]. Since *D. roeselum* microsporidia is a parasite that specifically infects female gammarids [7], this study was carried out on female gammarids only.

## Results

**Figure 1:**
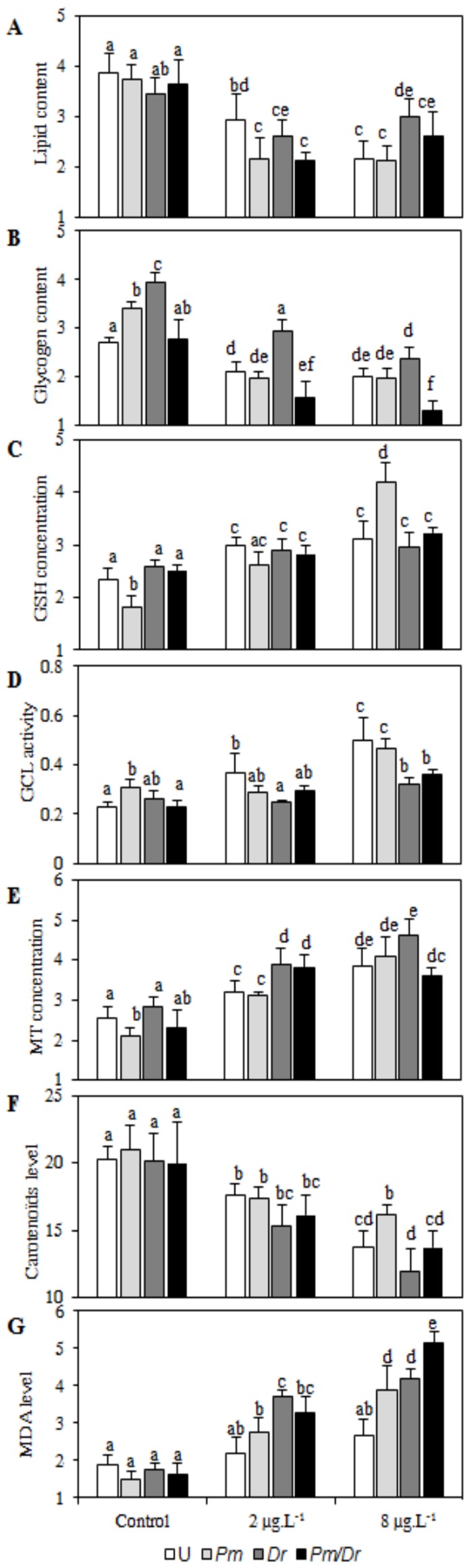
Influence of the presence of *P. minutus* and/or *D. roeselum* on energy and defences biomarkers of *Gammarus roeseli* females exposed to 2 and 8 µg Cd/L for 96 hrs. Pm: *P. minutus*-infected females, Dr: *D. roeselum*-infected females, Pm/Dr: bi-infected females. Different letters above the bars indicate significantly different values (Tukey's HSD test, *p*-values<0.05).

MANOVA results for the combined effects of infection and cadmium exposure on energetic reserves and antitoxic defences are given in Table 1. The two parameters had a general effect on the physiological condition of *G. roeseli* females, whether alone or in interaction. As predicted, the general outline was that cadmium exposure generally decreased gammarids' energetic reserves and increased stress marker levels (Figure 1). However, the extent of the changes depended on the cadmium dose and on the infection status, particularly in mono-infected individuals versus bi-infected ones.

**Table 1:** MANOVA and ANOVAs for the effects of parasite and cadmium on biomarkers.

Models	Parameters	Source of variation	*num d.f* §, *den d.f* †	*F*	*p-values*
**MANOVA**		Parasite	21, 168	12.65	**<0.0001** *
		Cadmium	14, 110	32.57	**<0.0001**
		Parasite×Cadmium	42, 354	6.28	**<0.0001**
**ANOVAs**	Total lipids	Parasite	3,60	3.18	**0.0302**
		Cadmium	2.60	83.64	**<0.0001**
		Parasite×Cadmium	6,60	5.82	**<0.0001**
	Glycogen	Parasite	3,60	92.68	**<0.0001**
		Cadmium	2,60	230.36	**<0.0001**
		Parasite×Cadmium	6,60	7.21	**<0.0001**
	GSH	Parasite	3,60	0.34	0.7955
		Cadmium	2,60	123.71	**<0.0001**
		Parasite×Cadmium	6,60	26.18	**<0.0001**
	GCL	Parasite	3,60	19.34	**<0.0001**
		Cadmium	2,60	88.72	**<0.0001**
		Parasite×Cadmium	6,60	8.2	**<0.0001**
	MT	Parasite	3,60	4.62	**0.0056**
		Cadmium	2,60	171.40	**<0.0001**
		Parasite×Cadmium	6,60	7.22	**<0.0001**
	Carotenoid	Parasite	3,60	7.26	**0.0003**
		Cadmium	2,60	101.32	**<0.0001**
		Parasite×Cadmium	6,60	1.61	0.1605
	MDA	Parasite	3,60	24.55	**<0.0001**
		Cadmium	2,60	209.73	**<0.0001**
		Parasite×Cadmium	6,60	12.43	**<0.0001**

* Significant values shown in bold.

§ Numerator degrees of freedom.

† Denominator degrees of freedom.

### 1. Low effects of the infection status in the absence of pollution

There was no significant impact of parasitic infection on three markers studied here (lipid, carotenoid and malondialdehyde levels, see Figures 1A, 1F & 1G). Glycogen contents strongly increased in response to each separate parasite species (Figure 1C) while in response to bi-infection their contents were similar to those of uninfected individuals. Finally, in response to mono-infection by *P. minutus*, reduced glutathione (GSH), γ-glutamylcysteine ligase (GCL) and metallothionein (MT) levels increased as compared to uninfected controls or to mono-infection by *D. roeselum*, but in response to bi-infection, the levels of these toxicity markers were restored to the level of uninfected individuals (Figures 1C, 1D & 1E).

### 2. Differential effects of cadmium exposures depending on the infection status

Except for carotenoid concentrations (Table 1), all biomarkers were impacted differentially by cadmium exposure depending on gammarid infection status.

Whatever the infection status, lipid contents were similarly affected by low-dose cadmium exposure. After high-dose cadmium exposure, *D. roeselum*-infected gammarids suffered less than uninfected females gammarids. Bi-infection did not accentuate the effect of cadmium exposure as compared to mono-infection (Figure 1A).

At the two cadmium exposures, bi-infection impacted negatively on glycogen contents as compared to mono-infections (Figure 1B). In mono-infected females, glycogen reserves approached those of uninfected ones, except for microsporidia-infected females at our low cadmium dose: in that case, glycogen reserves remained higher, as in the controls.

The effects of bi-infection on GSH and GCL were similar: while the low cadmium dose impacted all gammarids in a similar way, whatever their infection status, results were more contrasted at high cadmium dose (Figures 1C & 1D). In that case, while acanthocephalan mono-infection induced either a higher response (GSH) than in uninfected controls or a similar response (GCL), microsporidia mono-infection and co-infection induced similar responses to cadmium exposure.

The changes in metallothionein concentrations induced by cadmium exposure depended on both the infection status and the pollutant dose (Figure 1E). At low cadmium dose, the response of microsporidia-infected females was similar to that of bi-infected ones, and their response was higher than the response of uninfected or acanthocephalan-infected females. At high cadmium dose, the MT concentration pattern did not show such differences: bi-infected females displayed lower concentrations than microsporidia-infected ones.

For carotenoids, the higher the cadmium dose, the lower their concentrations, with a similar pattern for all infection statuses (Figure 1F). However, infection status also influenced on carotenoid contents: *D. roeselum*- and bi-infected females contained less carotenoids than acanthocephalan-infected females (Figure 1F).

Finally, malondialdehyde levels increased in all gammarids after cadmium exposure, whatever the infection status, but the phenomenon was more marked in bi-infected females at high cadmium dose (Figure 1G).

### 3. Integrated Biomarker Responses

Integrated Biomarker Responses (IBR hereafter) makes it possible to study differential responses between several conditions thanks to biomarker combinations. IBR was estimated to examine antitoxic defence responses (GSH, GCL, MT, carotenoid) and the toxicity effect (MDA) measured in the four infection statuses. As energy reserves can vary depending on gammarid physiological status, they were not included in the estimation.

**Figure 2:**
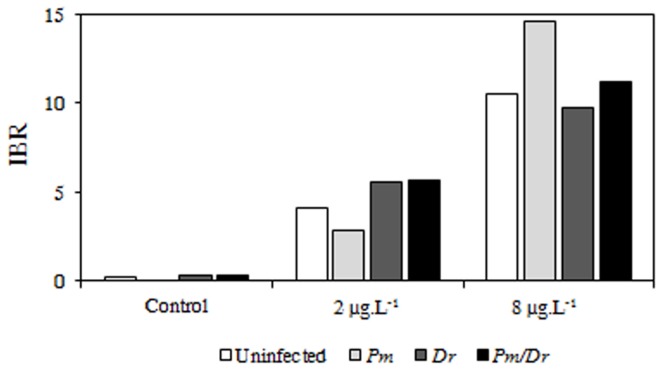
Integrated Biomarker Responses measured in *G. roeseli* exposed to 2 and 8 µg Cd/L for 96 hrs. Pm: *P. minutus*-infected females, Dr: *D. roeselum*-infected females, Pm/Dr: co-infected females. IBR values include defence responses (GSH, GCL, MT, carotenoids) and MDA responses.

The higher the stress, the higher the IBR value (Figure 2). Results revealed that gammarids were more stressed at 8 µg Cd.L^−1^ than at 2 µg Cd.L^−1^ whatever the infection status. However, it was observed that when they were exposed to the low cadmium concentration (2 µg Cd.L^−1^), *P. minutus*-infected females displayed the lowest stress levels, while they were also those displaying high stress levels when exposed to the highest cadmium concentration (8 µg Cd.L^−1^). At 2 µg Cd.L^−1^, *D. roeselum*-infected females and bi-infected females were more stressed than uninfected and *P. minutus*-infected ones. At the highest cadmium concentration, *D. roeselum*-infected females were slightly less stressed than uninfected ones, whereas bi-infected females were slightly more stressed than uninfected ones, but less than *P. minutus*-infected ones.

### 4. Malondialdehyde level in *P. minutus*

**Figure 3:**
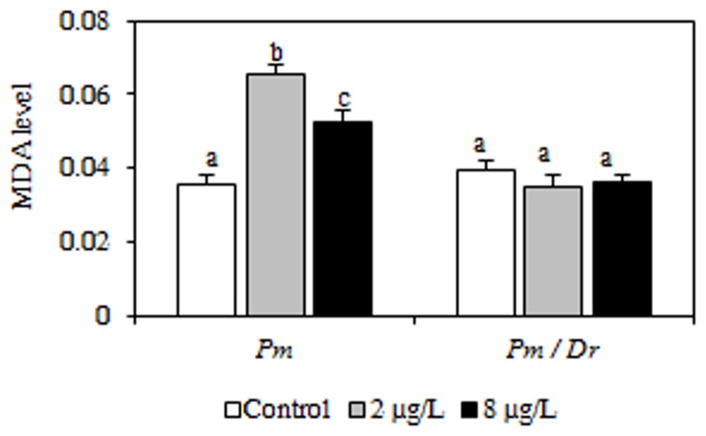
Malondialdehyde levels measured in the acanthocephalan parasite *P. minutus* dissected from *P. minutus*-infected *G. roeseli* females (Pm) and from *P. minutus*/*D. roeselum*-bi-infected females (Pm/Dr) exposed to 2 and 8 µg Cd/L. Different letters above the bars indicate significantly different values (Tukey's HSD test, *p*-values<0.05).

Malondialdehyde (MDA) levels were measured in *P. minutus* sampled either from *P. minutus*-infected or from bi-infected females. When *P. minutus* was the only parasite, it displayed increased MDA levels after cadmium exposure. However, when it shared its host with *D. roeselum*, no significant difference was observed between the three cadmium conditions (Figure 3, Table 2).

**Table 2:** ANOVA for the effects of host infection status and cadmium on the *P. minutus* malondialdehyde level.

Source of variation	Sum of Square	d.f	F	*p*-values
**Host infection status**	0.0012	1	128.04	**<0.0001**
**Cadmium**	0.0006	2	34.40	**<0.0001**
**Host infection status×Cadmium**	0.0012	2	64.47	**<0.0001**

## Discussion

The aim of the present study was to investigate the influence of bi-infection by the horizontally-transmitted parasite *P. minutus* and by the vertically-transmitted parasite *D. roeselum* on the energy reserves and the defence capacities of *G. roeseli* female gammarids after exposure to an additional stress, i.e. cadmium stress. Previous studies have demonstrated the influence of single parasites on their hosts under stressful conditions [7], [38], [39], but no investigations have examined the effect of the presence of two parasites sharing the same host on the energy reserves and antitoxic defences in a contamination context, especially parasites with different transmission strategies. The concomitant presence of *P. minutus* and *D. roeselum* clearly influenced the energy reserves and the defence capacities of *G. roeseli* females when they were exposed to cadmium, often in a way that contrasted with their response to mono- infection by the same parasite species.

First of all, it is worth noting that in the absence of pollutant stress, the two parasite species studied in this paper did not noticeably influence on the physiological parameters measured, except for the drop in glycogen contents in bi-infected hosts as compared to mono-infected ones (mono-infected hosts displayed higher glycogen contents than uninfected ones, as previously observed [7]), and for the lower reduced glutathione concentrations measured in *P. minutus*-infected gammarids. Such a global, relatively weak effect of parasitism on physiology has already been observed. It could enable the host to compensate for parasitic effects in non-stressful conditions [7]. In addition, results show that bi-infection did not increase the effects of parasitism on host physiology as compared to mono-infections.

Cadmium exposure dramatically changed that pattern. Only carotenoid levels decreased in the same way in all infection statuses after pollutant exposure. All other parameters changed differently depending on the cadmium dose and in interaction with the infection status. Two main categories of responses were observed. The first response type was an increased pathogenic effect in bi-infected hosts as compared to mono-infected ones. That response was observed on glycogen contents and, perhaps more importantly, on malondialdehyde (MDA) levels at the high cadmium dose. Since high MDA levels indicate high cell damage, the damage caused by bi-parasitism was therefore higher than the damage caused by each separate parasite. The second type of response was a similar response between *D. roeselum*-infected and bi-infected hosts. This was observed for all antitoxic defences: at all cadmium doses for carotenoids, at the high cadmium dose for reduced glutathione concentrations and γ-glutamylcysteine ligase activity, at the low cadmium dose for metallothionein concentrations. When we combined all antitoxic and damage biomarkers into a single index (IBR, Integrated Biomarker Response), the same pattern emerged: the response of bi-infected hosts was similar to that of *D. roeselum*-infected hosts and contrasted with that of *P. minutus*-infected hosts. Under low cadmium exposure, while *P. minutus*-infected gammarids displayed a weaker response to pollution as compared to uninfected ones, *D. roeselum*-infected and bi-infected gammarids displayed higher IBRs. Conversely, under high cadmium exposure, the IBR value was higher in *P. minutus*-infected gammarids than in uninfected, *D. roeselum*-infected and bi-infected ones. This combined response therefore indicates that the host's response to the acanthocephalan parasite was overridden by its response to microsporidian infection when the two parasites shared the same individual host. As infection by microsporidia always precedes infection by acanthocephalans due to the vertical transmission mode of *D. roeselum*, this could be interpreted as a way for the first established parasite to prevent a second, super-infecting parasite from potentially manipulating host physiology.

In cases of bi-infections, the precedence of the first established parasite has already been observed in different host-parasite systems (but see Lohr et al. [40] for a counter-example). For example, after its vertical transmission, the microsporidia *Octospora bayeri* was able to withstand competition with the bacterium *Pasteuria ramosa* in its host *Daphnia magna* to some extent, while *P. ramosa* prevented *O. bayeri* development when they both infected their host simultaneously [41]. However, in that case, parasite fitness suffered bi-infection and their virulence was increased when compared to single infections. Even when the two co-infecting parasites have the same transmission mode, similar findings have been evidenced. Thomas et al. [42] found that avirulent fungal entomopathogens could alter the virulence and the reproduction of a virulent one in the desert locust, depending on infection sequence and on environmental conditions. Similarly, Hughes and Boomsma [43] demonstrated that the avirulent fungus *Aspergillus flavus* sporulated better in the presence of the virulent fungus *Metarhizum anisoplia* in their leaf-cutting ant host. Finally, numerous vertically-transmitted symbionts (mainly bacteria) protect their hosts from infection by horizontally-transmitted parasites, for example parasitoids, viruses or *Plasmodium* (see Haine [44] for a review, and Moreira et al. [45] for an additional example).

In the host-parasite system studied here, the vertically-transmitted microsporidia did not protect gammarids from acanthocephalan infection, but increased their tolerance to it. Indeed *P. minutus* induces changes in the *G. roeseli* host's behaviour that favour its transmission to the next host but also lead to the death of the gammarid host after the predation by the next host [20], [24], [46]. These behavioural changes, added to the castration that *P. minutus* also induces in *G. roeseli* [14], are unfavourable for vertically-transmitted microsporidia. Haine et al. [14] found that behavioural manipulation by *P. minutus* was weaker in *D. roeselum*-co-infected *G. roeseli* than in *G. roeseli* infected by *P. minutus* alone.

The results described here (namely a same pattern of antitoxic responses in *P. minutus*/*D. roeselum*-bi-infected *G. roeseli* as in *D. roeselum*-infected *G. roeseli*) are in line with those of Haine et al. [14]: it appears that most of the effects of *P. minutus* were cancelled by the presence of the vertically-transmitted microsporidia. We can hypothesise that *D. roeselum* could alter the influence of *P. minutus* on *G. roeseli* defence capacities, which could reduce the horizontally-transmitted parasite to a kind of “silent parasite”. In a previous study, we showed that *P. minutus*, when the only parasite present, accumulated cadmium after the exposure of its *G. roeseli* host [47]. This accumulation causes cell damage in the parasite, a result confirmed in the present study. However, we found here that cadmium exposure did not increase MDA levels in *P. minutus* when its host was already infected by *D. roeselum*. The acanthocephalan therefore suffered less cell damage in these conditions. The interference between the two parasites could be due to *D. roeselum* reducing the exchanges between *P. minutus* and the host (e.g. nutrients exchanges), reducing cadmium accumulation in *P. minutus* and thus reducing cell damage. Yet our result unveil a paradox since on the other hand the interaction between the two parasites clearly resulted in increased cell damage in the host (relative to mono-infections), which could be interpreted as an increase of virulence due to bi-infection.

In conclusion, this work is the first study investigating the influence of a bi-parasitism on the energy reserves and antitoxic defences of a host in a contamination context. Bi-infections resulted in higher cell stress and lower glycogen contents than mono-infections in the host, only under additional pollutant stress. These two cumulative effects could be seen as a higher virulence due to bi-infection and could accentuate the decrease in host fitness in a contamination context. Nevertheless, the negative effects of bi-infection are not always cumulative, as shown by antitoxic defence responses. For these traits, the pattern of bi-infected host reaction to pollutant stress followed the pattern of microsporidia-infected hosts, i.e. the antitoxic response decreased most of the time (carotenoids, reduced glutathione concentrations and γ-glutatmylcysteine ligase activity), but it also increased depending on the cadmium dose (metallothionein concentration at our low cadmium dose). This pattern indicates that host physiology (and perhaps also acanthocephalan physiology, as suggested by our results) can be controlled to some extent by microsporidia under stressful conditions. This result is in line with the sabotage effect of this parasite on host behavioural manipulation by acanthocephalan [14]. Globally, however, since microsporidia often decrease their host's antitoxic defences, and therefore the host's capacity to cope with pollutant stress, such control by microsporidia is not beneficial to the host in a contamination context. Such a global negative effect of bi-parasitism on host condition should of course be tested on more biological models. As noted by Jones et al. [48] in a theoretical model, competition between parasites critically depends on the life history trade-offs of the two parasites as these trade-offs may be highly specific, especially when considering horizontally-transmitted and vertically-transmitted parasites.

## Materials and Methods

### 1. Sampling collection, maintenance and cadmium exposure

*P. minutus*-uninfected and naturally infected *G. roeseli* females were collected in April 2011 using pond nets in the French Nied River (Rémilly, North-eastern France, 49° 00′N and 6° 23′E), where cadmium concentrations were less than 0.2 µg.L^−1^ (LADROME laboratory, Valence, France). *P. minutus* parasites were easily identified in the field since the cystacanth stage appears as an intense orange dot through the cuticle, while microsporidian parasites *D. roeselum* are mainly localized in the gonadal tissues and are therefore “hidden” from observers. Experiments were therefore made in blind for *D. roeselum* infection: detection was carried out afterward (see 2. for methods). All gammarids females with signs of septicemia, due to the presence of bacteria or fungi, were excluded from analyzes. We sampled 600 females gammarids infected by *P. minutus* and 600 females gammarids uninfected by it. Female gammarids were sorted in the field thanks to gnathopod size, which is a sexual dimorphism character. They were transferred to the laboratory in large containers filled with river water, acclimated 5 days at 15°C in EDTA-free Elendt M4 solution, and fed *ad libitum* with alder leaves.

Test solutions were prepared using EDTA-free Elendt M4 solution with CdCl_2_ added to obtain three cadmium conditions: 0, 2 and 8 µg Cd.L^−1^. These concentrations were defined according to Gismondi et al. (2012) [7]. The two sets of females gammarids (infected by *P. minutus* or not) were divided into three sub-sets, each exposed at 15°C for 96 hrs at one cadmium concentration in aquaria previously saturated for 5 days to avoid cadmium adsorption. After the exposure, the gonadal tissue of each individual was dissected and stored at −80°C awaiting DNA extraction. In addition, in *P. minutus*-infected series, the acanthocephalan was removed and stored at −80°C awaiting marker analysis. All gammarids were individually frozen in liquid nitrogen and stored at −80°C awaiting biomarker analysis. The dissection step also allowed us to exclude the individuals infected by other macro-parasites (e.g. cestodes, nematodes, trematodes).

### 2. Determination of *D. roeselum* presence

After DNA extraction from the gonads, the microsporidian status of each *G. roeseli* (i.e. presence or absence of *D. roeselum*) was determined using a PCR-restriction fragment length polymorphism (PCR-RFLP) method, as described in Haine et al. (2004) [30]. The PCR products (∼500 pb) were digested by the restriction enzymes *Bst1107I* following the manufacturer's instructions (MBI Fermentas) in order to identify *D. roeselum* specifically. Four different groups (thereafter called infection status) were then established: (i) uninfected females (U females), (ii) *P. minutus*-infected females (Pm females), (iii) *D. roeselum*-infected females (Dr females) and (iv) bi-infected females (Pm/Dr females). A few females were found infected by another vertically-transmitted microsporidia (*Dictyocoela muelleri* - Dm, see Gismondi et al. [7]). Since the sample size was too small to allow a statistical analysis of biomarker results (n = 21 for Pm/Dm females and n = 48 for Dm-infected females), they were removed from further analyses.

### 3. Biomarker measurement

Assaying antitoxic defences is impossible to perform on individual gammarids, a minimum number of six gammarids being necessary to have enough tissues to analyse. The number of replicates was therefore calculated from the minimal sample size obtained for a given infection status. The lower sample size obtained was n = 40 for Dr females exposed to 8 µg Cd.L^−1^. Therefore, for each exposure condition, 6 replicates of 6 individuals with the same infection status were made to measure energy reserves and antitoxic defences as described below. For series with large sample size (e.g. uninfected females) the 36 animals used for the measures were taken at random.

#### 3.1 Sample preparation

Each replicate was homogenized with a manual Potter Elvejhem tissue grinder in 50 mM phosphate buffer KH_2_PO_4_/K_2_HPO_4_ (pH 7.6) supplemented with 1 mM phenylmethylsulphonylfluoride (PMSF) and 1 mM L-serine-borate mixture as protease inhibitors, and 5 mM phenylglyoxal as a γ-glutamyl transpeptidase inhibitor. The homogenization buffer was adjusted at a volume two-fold the wet weight of the sample pool (e.g. 200 µL of homogenization buffer for 100 mg of wet weight tissue). The total homogenate was divided into seven parts to measure the different parameters. For each replicate, two independent measures were made for each biomarker.

#### 3.2. Energy reserves

The measurement of total lipid and glycogen contents was adapted from Plaistow et al. (2001) [49]. Twenty microlitres of 2% sodium sulphate (w/v) and 540 µL of chloroform/methanol 1∶2 (v/v) were added to 40 µL of the total homogenate. After 1 hr on ice, the samples were centrifuged at 3,000× g for 5 min at 4°C. The resulting supernatant and the pellet were used to determine the total lipid and glycogen contents, respectively.

One hundred microlitres of the supernatant were transferred into culture tubes and placed in a dry bath at 95°C to evaporate the solvent. Then, 200 µL of 95% sulphuric acid were added in each tube and left for 10 min. The culture tubes were cooled in ice and then 4.8 mL of phosphovanillin reagent were added. After a 10-min reaction, the optical density was measured at 535 nm. Commercial cholesterol was used as a standard and total lipid contents were expressed in mg.mL^−1^.

Total dissolution of the pellet was performed in 400 µL of deionized water for 10 minutes in an ultrasonic bath. One hundred microlitres of each sample were placed into culture tubes and 4.9 mL of Anthrone reagent were added. The mixture was placed in a dry bath at 95°C for 17 min and then cooled on ice. Optical density was measured at 625 nm. Glucose was used as a standard and concentrations were expressed in µg.mg^−1^ tissue.

The total protein content of each sample was quantified according to Bradford (1976) [50] with bovine serum albumin (BSA) as a standard. Results were expressed in mg.mL^−1^.

#### 3.3. Antitoxic defence capacities

Reduced glutathione (GSH) concentration measurement was adapted from Leroy et al. (1993) [51] using High-Pressure Liquid Chromatography (HPLC) separation. The proteins of 40 µL of the total homogenate were precipitated with 10% perchloric acid (v/v). After a 10-min centrifugation at 20,000× g at 4°C, the resulting supernatant was diluted 40-fold in 0.1 M HCl. Commercial GSH diluted in 0.1 M HCl was used as a standard and GSH concentrations were expressed in nmol GSH.mg^−1^ protein.

GCL activity was assayed using an HPLC method adapted from Parmentier et al. (1998) [52]. Measurements were carried out on the S12000 fraction obtained after centrifuging 40 µL of the total homogenate for 15 min at 500× *g* and then centrifuging the resulting supernatant at 12,000× g and 4°C for 30 min. The resulting S12000 fraction was diluted 20-fold in the homogenization buffer and 40 µL of this diluted solution were added to 112 µL of incubation cocktail (0.5 M Tris-HCl, 200 mM MgCl_2_,6H_2_O, 500 mM KCl, 45 mM glutamic acid, 90 mM cysteine, 1 mM DTT, 90 mM ATP, 0.5 mM phenylglyoxal, pH 8.25) to initiate the reaction. After a 20 min-incubation period at 25°C, the reaction was stopped by a four-fold dilution with 0.1 M HCl. Commercial glutamylcysteine (GC) solution prepared in 0.1 M HCl was used as a standard and GCL activity was expressed in nmol GC.min^−1^.mg^−1^ protein.

Metallothionein (MT) concentrations were determined with an HPLC method adapted from Alhama et al. (2006) [53]. Forty microlitres of the total homogenate were centrifuged at 3,500× g for 10 min. The resulting supernatant was centrifuged at 22,000× g for 30 min at 4°C to obtain the S22000 fraction. A ten-fold dilution of the S22000 fraction was prepared in 100 mM Tris buffer (pH 9.5) supplemented with 1 mM DTT and 100 mM PMSF as a protease inhibitor. To reduce and denature the protein, 125 µL of diluted sample were added to 108 µL of incubation cocktail (230 mM Tris pH 9.5, 300 mM DTT, 100 mM EDTA and 10% sodium dodecyl sulfate) in a water bath at 70°C for 20 min. Then, the incubation mixture was supplemented with 17 µL of 180 mM monobromobimane (mBBr) and incubated in the dark at room temperature for 15 min, to mark MTs. Commercial rabbit-liver MT I solution prepared in 230 mM Tris, pH 9.5, was used as a standard and MT concentrations were expressed in nmol MT.mg^−1^ protein.

Carotenoïd concentrations were measured by a spectrophotometry method adapted from Rauque and Semenas (2009) [54]. Fifty microlitres of the total homogenate were diluted in 450 µL of ethanol 96% and kept 6 hrs in the dark at 4°C, before being centrifuged 10 min at 3,500× g. The optical density of the resulting supernatant was measured at 422, 448 and 476 nm using a commercial mixture of carotenoïds as a standard. Carotenoïd concentrations were expressed in ng carotenoïds.mg^−1^ lipid.

Malondialdehyde (MDA) levels were measured using an HPLC method adapted from Behrens and Madère (1991) [55] with UV detection at 267 nm. Seventy microlitres of the total homogenate were diluted four-fold with 95% ethanol (HPLC grade) and cooled on ice for 1.5 hrs to deproteinise them. The mixture was then centrifuged at 18,000× g for 30 min at 4°C. One hundred microlitres of the resulting supernatant were injected into the HPLC separation system. MDA levels were expressed in ng MDA.mg^−1^ lipid.

For MDA measurements in *P. minutus*, four replicates of 5 *P. minutus* cystacanths were carried out according to the infection status of their respective hosts (e.g. mono- or bi-parasitism), for each cadmium condition. Each replicate was crushed in 350 µL of ethanol 95% and kept 1.5 hrs on ice. The measurement was then performed as described above.

### 4. Integrated Biomarker Response

To obtain a global view of the effect of the parasite/cadmium interaction, an Integrated Biomarker Response (IBR) value was estimated according to the method of Beliaeff and Burgeot (2002) [56]. Only antitoxic defence responses were taken into account in our IBR estimation, which is briefly described here. The mean (*m*) and the standard deviation (*s*) of each biomarker were calculated for all exposure conditions; then, data were standardised for each exposure condition:


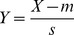


where *Y* is the standardised value and *X* is the mean of each biomarker for each exposure conditions. Standardised values make it possible to estimate *Z*, which is equal to *+Y* when biomarkers increase and to *−Y* when they decrease. Then, the minimum value for all exposure condition of each biomarker was obtained, and was added to *Z*, to obtain the score *S*:


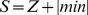


Finally, the IBR value for each exposure condition was determined, thanks to the following formula, where n is the number of biomarkers:


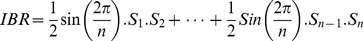


### 5. Statistical analysis

Our data met normality and variance homogeneity conditions (Shapiro and Bartlett tests, *p*>0.05). Comparisons were performed by using an ANOVA test, followed by a post-hoc HSD Tukey test.

All tests were performed using R 2.9.0 and were two-tailed with significant differences considered at the level of *p*-values<0.05.
